# The effect of nest temperature on growth and survival in juvenile Great Tits *Parus major*

**DOI:** 10.1002/ece3.7565

**Published:** 2021-05-01

**Authors:** Alejandro Corregidor‐Castro, Owen R. Jones

**Affiliations:** ^1^ Department of Biology University of Southern Denmark Odense M Denmark; ^2^ Interdisciplinary Center on Population Dynamics (CPop) University of Southern Denmark Odense M Denmark

**Keywords:** climate change, juvenile development, reproductive success

## Abstract

1. For birds, maintaining an optimal nest temperature is critical for early‐life growth and development. Temperatures deviating from this optimum can affect nestling growth and fledging success with potential consequences on survival and lifetime reproductive success. It is therefore particularly important to understand these effects in relation to projected temperature changes associated with climate change.
2. Targets set by the 2015 Paris Agreement aim to limit temperature increases to 2°C, and, with this in mind, we carried out an experiment in 2017 and 2018 where we applied a treatment that increased Great Tit *Parus* *major* nest temperature by approximately this magnitude (achieving an increase of 1.6°C, relative to the control) during the period from hatching to fledging to estimate how small temperature differences might affect nestling body size and weight at fledging and fledging success.
3. We recorded hatching and fledging success and measured skeletal size (tarsus length) and body mass at days 5, 7, 10, and 15 posthatch in nestlings from two groups of nest boxes: control and heated (+1.6°C).
4. Our results show that nestlings in heated nest boxes were 1.6% smaller in skeletal size at fledging than those in the cooler control nests, indicating lower growth rates in heated boxes, and that their weight was, in addition, 3.3% lower.
5. These results suggest that even fairly small changes in temperature can influence phenotype and postfledging survival in cavity‐nesting birds. This has the potential to affect the population dynamics of these birds in the face of ongoing climatic change, as individuals of reduced size in colder winters may suffer from decreased fitness.

## Graphical abstract

Nest temperature has an important effect on the early stages of bird development and could influence fledging success and growth with longer‐term consequences. We increased Great Tit nest temperature by 1.5°C (temperature limit increase set by 2015 Paris Agreement). Our results show that nestlings in heated nest boxes were 1.6% smaller in size at fledging than those in the cooler control nests, indicating lower growth rates in heated boxes, and that their weight was, in addition, 3.3% lower.

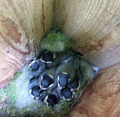

## 1 INTRODUCTION

Environmental conditions during early development are well known to play an important role in shaping an organism's phenotype (Naguib et al., 2011), because these early conditions can have long‐lasting cross‐generational effects (Monaghan, 2007; Naguib et al., 2011). Several authors have addressed the influence of temperature in early development and have found effects on short‐ and long‐term phenotypic expression, such as survival, growth, and size (Andrews et al., 2000; Bourne et al., 2020; Shine et al., 1997; Van Damme et al., 1992).

Body size is one of the most important phenotypic traits because it is a well‐known determinant of survival and breeding success of wild animals. Of crucial importance, therefore, is the body size achieved at independence or sexual maturity. In general, smaller individuals will fare less well and have lower lifetime reproductive success than those of a larger size (Bolton, 1991; Cox et al., 2014; Garnett, 1981; Sullivan, 1989).

One adaptation to temperature regimes is the reduction of body size, as small body sizes will increase the surface‐area‐to‐volume ratio, and thereby exacerbate heat loss (Teplitsky et al., 2008). Yom‐Tov (2001) tackled phenotypic plasticity in a long‐term study (1950–1999) in several bird species and showed that some of them, such as the House Sparrow *Passer* *domesticus*, reduced both their average body mass and tarsus length as average ambient temperature increased, and these changes were likely due to phenotypic plasticity rather than selection (microevolutionary change) (Teplitsky et al., 2008). Although such observational approaches are powerful, they cannot easily disentangle the role of temperature from other potential drivers that may also be correlated with temperature such as food availability (Burguer et al., 2012; Vedder et al., 2013).

Future climate scenarios include changes to local temperature regimes, and therefore, a timely question is: If temperature does influence adult body size, how exactly is this mediated? The effects of these temperature changes may be perceived in the early stages of the bird's life, where the thermal environment of the nest plays an important role in determining the energetic investment of the young (Rodríguez & Barba, 2016a). Here, we use an experimental approach using a population in artificial nest boxes where we can easily monitor our study species. Specifically, we aim to quantify the role of temperature during a critical stage of the altricial Great Tit *Parus* *major* development—the nestling period (i.e., between hatching and fledging). Upon hatching, the chicks are naked, weighing approximately 1.5 g (Orell, 1983). They then grow rapidly, reaching their adult size and weighing around 16.0 g when they fledge (Orell, 1983).

We hypothesize that an experimentally increased nest temperature could result in a reduced adult skeletal size. Support for this hypothesis would demonstrate the important role of phenotypic plasticity rather than viability selection in determining body size variation within species. In investigating body size, we focus primarily on skeletal size (tarsus length) rather than body mass because measurements of the latter are particularly sensitive to food intake variability (Rising & Somers, 1989) and will consequently be more weakly related to growth and development processes compared with more robust measures.

## 2 MATERIAL AND METHODS

### 2.1 Study area

We conducted this study from February to June in 2017 and 2018 on a Great Tit population breeding in two areas of mixed temperate woodland in northern Europe around University of Southern Denmark's (SDU) Odense campus (55.372°N, 10.424°E). These woodlands are composed mainly of Sycamore *Acer* *pseudoplatanus*, Common Ash *Fraxinus* *excelsior*, and Wych Elm *Ulmus glabra* and are lightly managed by selective logging practices that remove trees that endanger safety of walkers.

### 2.2 Nest boxes and experimental treatment

In 2013, 100 nest boxes were deployed as part of the SDU Bird Project aimed to monitor the Great Tit's population biology in the area. These nest boxes are made of pine, with 2 cm thick walls and the entrance hole located 18 cm above the bottom of the box. They have a rectangular shape with a volume of approximately 6.5 L (23.5 cm height and a 14 × 19.6 cm base; Appendix S1, Figure S1). As a maintenance procedure, and according to Lambrechts et al. (2010), the nest boxes are cleaned before the beginning of each nesting season (by the end of February) to remove winter nests from other birds or mammals and to reduce the potential ectoparasite load (Goodenough et al., 2011). Following the recommendations of Lambrechts et al. (2010) for a better occupancy, the nest boxes are secured against the tree trunks with wire ~1.5 m above the ground. For this study, we ensured that every nest box faced southeast. This is important because previous studies have revealed an effect of orientation on various measures including occupancy, nestling body mass, and microhabitat (Goodenough et al., 2008, 2011; Goodenough & Stallwood, 2012). By facing all the nest boxes in the same orientation, the confounding effects of orientation were reduced.

Before the breeding season began, we prepared all of the nest boxes to allow artificial heating of their chambers, aiming to increase temperatures within the nest box by approximately 2°C (Appendix S1, Figure S1). We used this target treatment temperature increase because it is just beyond the target of a 1.5°C global increase set by the 2015 Paris Agreement (UNFCCC, 2015). We modified the nest boxes to allow the addition of insulation (polystyrene) and a heating source (UniHeat 72‐hr chemical heat packs) under the nest cup. This modification consisted of a cut 6 cm above the base acting as a removable door, where we inserted a piece of polystyrene with a thickness of 2 cm. This avoided direct contact with the nest itself, reducing the stress on the individual birds occupying the nest box and preventing them from leaving the nest during the visits. The placement of the polystyrene insulation left a chamber below the nest where we placed the heat packs. Over this material, we placed a water‐resistant piece of wood with a thickness of 3 mm to protect the polystyrene from weathering. There was about 1 cm between the heat pack and the insulation layer, so the heat pack could function properly, due to its need for oxygen to start the chemical reaction (by oxidizing iron powder).

### 2.3 Nest temperature and ambient weather

To document temperatures in the heated and control nest boxes, we used wire to securely anchor DS1921G Thermochron iButton temperature data loggers (Maxim Integrated, accuracy ± 0.5°C) immediately adjacent to the nest material cup of a subset of our nest boxes (2017, *n* = 14; 2018, *n* = 24, divided equally among heated and control treatment groups). The position of the iButton ensured that it was close to, but not touching, the nestlings (Appendix S1, Figure S2). We set the data loggers to record temperature every 5–10 min during the study period (13th–27th May 2017; 22nd–27th May 2018). To record ambient weather conditions, we obtained maximum and minimum daily temperatures from a nearby (5.46 km distance) weather station in Aarslev (55.317°N, 10.433°E). We obtained the data via the Global Surface Summary of the Day (GSOD) data provided by the US National Centers for Environmental Information (NCEI).

### 2.4 Monitoring of reproductive success and chick growth

From the beginning of March, we visited every nest box at least once per week until the beginning of April, when the survey frequency increased to every 2 or 3 days. This allowed us to estimate accurately the date of laying of the first egg, the first day of incubation, and the final clutch size, assuming that one egg was laid per day (Encabo et al., 2001). When incubation started (hereafter referred to as incubation day 1), we stopped the daily visits until incubation day 10, to reduce disturbance to the parents.

We determined the exact hatching date (hereafter referred to as hatching day 0) by daily inspections from day 11 after the beginning of incubation. On hatching day 1, we placed the first heat pack in the nest box, and we continued the nest visits every second day until 13 days posthatching, in order to replace the heat packs in the nest boxes under the heating treatment and to ensure an equal amount of disturbance to the control boxes. We placed used (nonactive) heat packs in the heating chamber of the control nest boxes. Unlike Álvarez and Barba (2014), we decided to replace the heat packs every 2 days and not three because, according to our earlier pilot study, the heat packs showed a marked decrease in their heat production capability after 48 hr. We alternately assigned each box to the “heated” or “control” group, depending on their hatching date and location in the forest. This stratified approach ensured that we avoided bias in temperature differences between groups due to measurements on different developing periods, or habitat factors such as nest density.

We ringed each nestling using an individually numbered metal ring between days 7 and 10 depending on the developmental status of the nestling (very small chicks were not ringed until they had a tarsus length of approximately 15 mm in length). We identified each nestling from day 5 until they were ringed by marking them on the skin of their vent and lower wing, and bill, with a nontoxic marker pen (Sharpies). We recorded the number and identity of nestlings alive on days 5, 7, 10, and 15 after hatching, and measured their tarsus length with a digital calliper (RS Pro; accuracy ± 0.01 mm) in order to obtain information on adult size of nestlings, measured as tarsus length at day 15 (Noordwijk, 2012), and weighted them with an electronic balance (accuracy ± 0.1 g). On day 20, we visited each nest box to confirm fledging and to measure fledging success, and we identified and counted dead individuals. For this experiment, we only used nestlings from first broods.

Our method allowed us to estimate the date of hatching of the first egg and the length of the incubation period. In addition, we recorded a suite of nest‐specific breeding parameters: clutch size, number of hatched, and fledged nestlings (and consequently probabilities of hatching and fledging). We obtained data from 30 nest boxes in 2017 (17 control, 13 heated) and 36 nest boxes in 2018 (17 control, 19 heated). The differences in sample sizes among treatments and years were due to varying occupancy of the modified boxes by other birds' species present in the area (i.e., Blue Tit *Cyanistes caeruleus*; Marsh Tit *Poecile palustris*; Eurasian Nuthatch *Sitta europaea*).

### 2.5 Statistical analyses

To test whether temperature had an effect on the breeding outcome and nestling size at day 15, we used GLMs including the treatment and the year (and their interaction). Because our heating treatment was applied only from the day of hatching onwards, we accounted for the potential confounding effect of prehatching variables. These prehatching variables, hereafter referred to as breeding parameters, which are fundamental in monitoring the breeding process, were the duration of incubation (in days), clutch size, and the brood size. We first standardized these values by subtracting the mean and dividing by the year‐specific standard deviation. Then, to determine if these potential confounding variables influenced our results, we fitted pairs of models with one including the potential confounding variable and the other omitting it. We tested the significance of the confounding variable using ANOVA to compare models. In addition, we controlled for among‐nest differences in the timing of reproduction by adding the day of hatching (again, standardized by subtracting the mean and dividing by the year‐specific standard deviation) as a covariate.

In summary, we fitted two models to test for treatment differences in the breeding outcome (hatching and fledging success), and two models to test for nestling size and weight at day 15 (tarsus length and body weight). All the statistical analyses were carried using the statistical software R version 4.0.1 (R Core Team, 2020).

### 2.6 Effects on the breeding outcome

We used hatching and fledging success as measurements of the breeding outcome. While the treatment was applied after hatching, we still decided to include the hatching success in the analysis, to further test for (and discard) initial bias. For the different sets of models for each of the variables, we followed the previously explained model comparison approach. For tractability, we conducted our analysis at the level of the nest rather than individual chick level. In each case, our response variable consisted of two values: number of successes (survival) and number of failures (i.e., death), and so our models were fitted with a binomial error structure.

### 2.7 Post‐treatment effects on nestling size and weight

In addition to our examination of the breeding outcome, we studied the effect of the temperature treatment on nestling size and weight. To do this, we graphically examined the brood mean tarsus length and body weight of our treatment groups (control and heated) on days 5, 7, 10, and 15 posthatching. Although it would have been interesting to fit growth‐curve models, such as Gompertz or von Bertalanffy functions (von Bertalanffy, 1957; Ricklefs, 1967), we did not have sufficient data points per individual to model such curves reliably. Instead, we chose to examine the size and weight achieved at day 15 after hatching, which is when these birds are known to reach adult skeletal size (Noordwijk, 2012). As explained above, we conducted a model comparison approach using GLMs with Gamma error structures.

## 3 RESULTS

**FIGURE 1:**
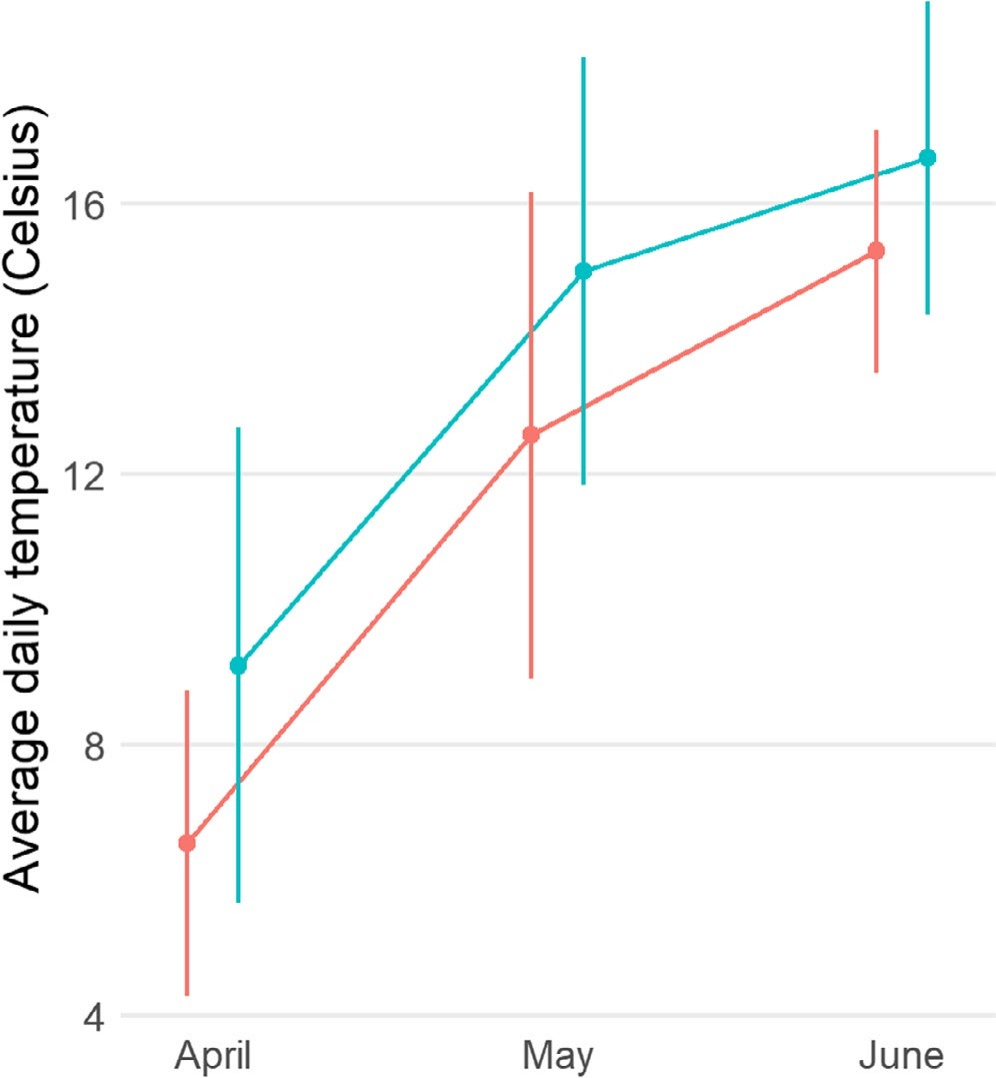
Average daily temperature (±1 standard deviation) in April–June 2017 and 2018. Red points/bars represent 2017, and blue points/bars represent 2018. Weather data obtained from a nearby weather station (Aarslev, 5.46 km away) via the Global Surface Summary of the Day (GSOD) data provided by the US National Centers for Environmental Information (NCEI)

Our comparison of nest temperature within a subset (*n* = 38) of heated and control nests showed that heated nests were, on average, 1.63°C warmer than control nests (i.e., around a maximum of 36.0°C versus 34.3°C during the day and dropping to 29.8°C versus 27.8°C during the night, see Appendix S1, Figure S3). Ambient air temperature tended to be cooler in 2017 than 2018 and increased from around 6.6 ± 2.3°C in April to 15.3 ± 1.8°C in June in 2017 and from 9.2 ± 3.5°C in April to 16.7 ± 2.3°C in June 2018 (mean ± *SD*; see also Figure 1).

### 3.1 Effects on the breeding outcome

**FIGURE 2:**
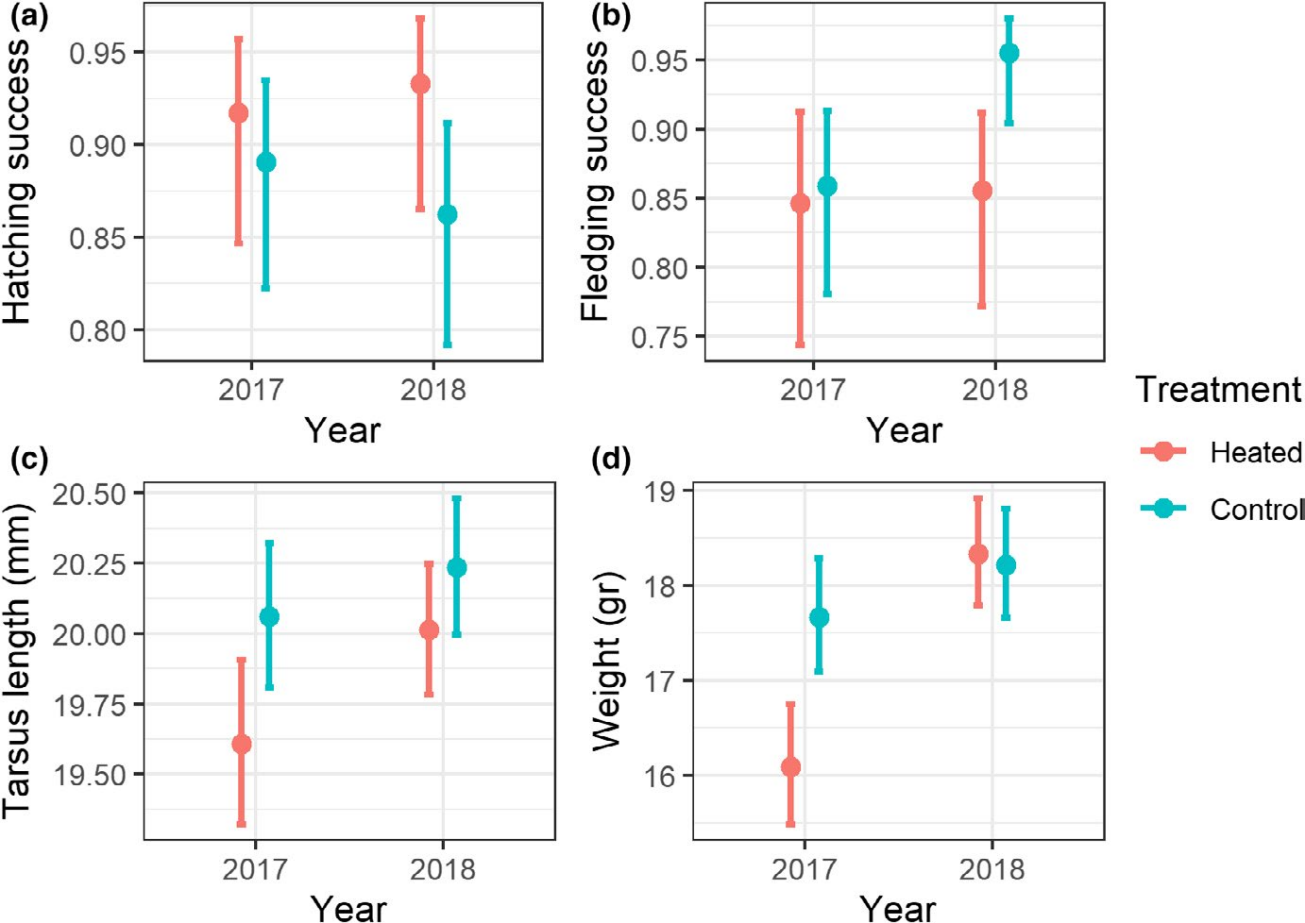
Results for the model prediction for each of the breeding outcome variables (a: hatching success; b: fledging success), as well as the tarsus length (c) and weight (d) at day 15. In red, expected results for an individual from the “heated” treatment, and in blue for an individual of the “control” treatment

Our model comparison showed that the treatment (“heated” vs. “control”) did not have a significant effect on breeding outcome in either year (2017, 2018), neither did it influence hatching success (*t*_5,58_ = 101.53, *p* = 0.47) or the fledging success (*t*_4,59_ = 176.60, *p* = 0.80). However, we note that in 2018, the effect of the treatment on the fledging success was larger on heated boxes, with slightly lower success (Figure 2b).

### 3.2 Post‐treatment effects on nestling size and weight

**FIGURE 3:**
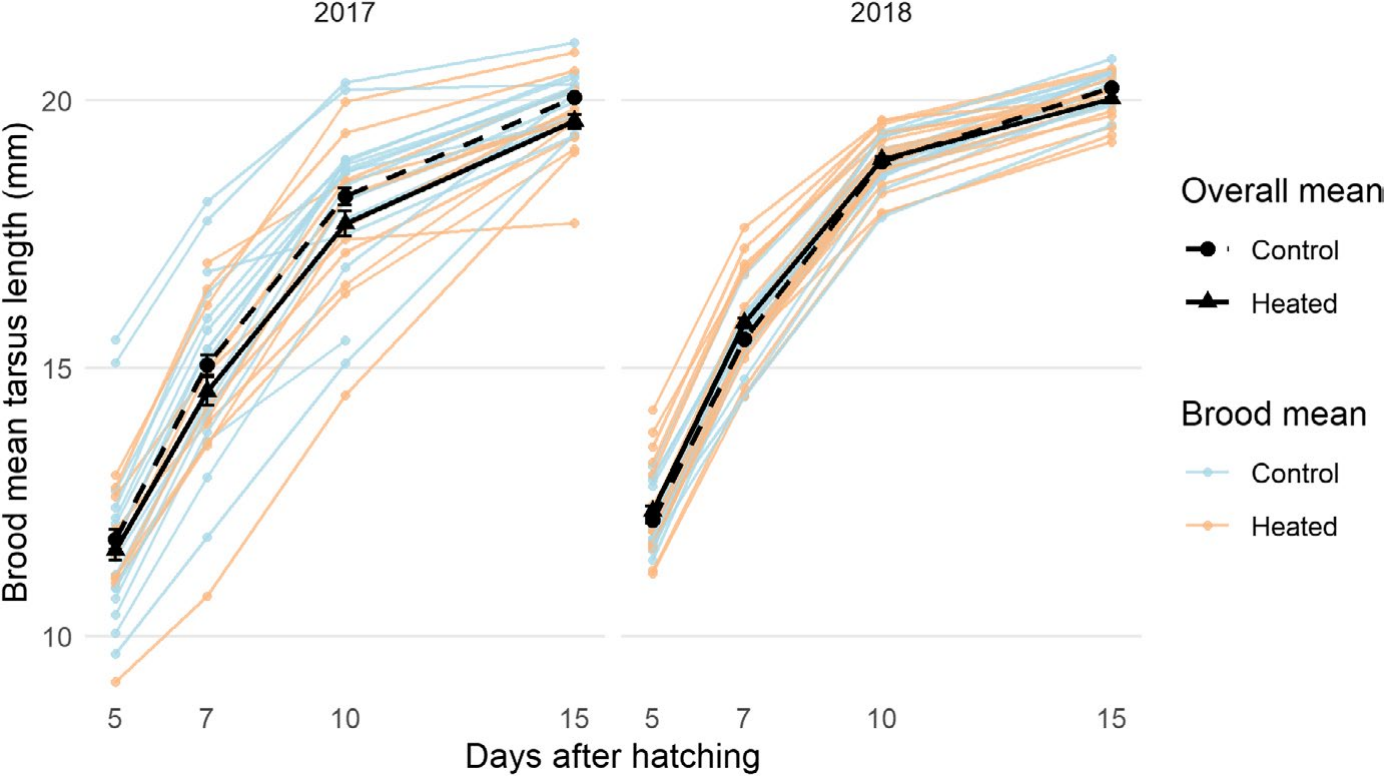
Brood mean tarsus length (mm) of both treatment groups during the two‐year period of study. The bold black lines indicate overall treatment means, while the pale colored lines indicate the brood‐level means which are shown to indicate variation. In both years, the average final tarsus size of the individual (day 15) is statistically significantly reduced in the heated group. Error bars on the overall mean points represent standard error and are in some cases obscured by the points

A visual inspection of the raw data (Figure 3) revealed that growth trajectories were broadly similar between years, but that there was greater variation in 2017 than in 2018. In addition, there was a clear and consistent difference in mean tarsus length in 2017, with the heated group being smaller than the control. In contrast, the differences between treatment groups were more ambiguous in 2018.

We found a significant effect of the treatment on both tarsus length (*t*_3,57_ = 0.04, *p* = 0.027) and body weight (*t*_3,57_ = 0.04, *p* < 0.001) at day 15. The model's population‐level estimates for tarsus length, adjusted for day of hatching, were 20.18 mm (95% CI = 19.97–20.39 mm) and 19.87 mm (95% CI = 19.67–20.08 mm) for control and heated groups, respectively: This was a 1.55% difference in size (Figure 2c). The estimates for the values of body weight were 18.02 g (95% CI = 17.02–19.03 g) and 17.42 g (95% CI = 16.40–18.41 g) for control and heated groups, respectively: This was a 3.34% difference in body weight among treatments (Figure 2d).

## 4 DISCUSSION

In this study, we carried out a field experiment investigating growth patterns in Great Tits and, in particular, the influence that nest temperature (and therefore thermoregulatory costs) have on adult size. We used tarsus length as our main indicator of adult size, because this measurement remains constant throughout adult life (Garnett, 1981) and is not as influenced by spatio‐temporal variation in environmental conditions as body weight. Nevertheless, we carried out an additional analysis using body weight which produced qualitatively similar results. We found that nestlings from warmer nests tend to reach a smaller skeletal size (as measured by tarsus length) than those in the cooler nests. Broadly, our study thus supports previous experimental findings (Rodríguez & Barba, 2016b; Rodríguez et al., 2016; Salaberria et al., 2014) and the extensive observational work using long‐term population data that have also found reduced body size with increasing temperature (McNab, 1971; Teplitsky et al., 2008; Weeks et al., 2019; Yom‐Tov, 2001).

A visual inspection of growth trajectories (Figure 3) shows that they followed a similar pattern in both years. However, although mean size was consistently smaller in the heated group in 2017 throughout the growing period, the pattern was more ambiguous in 2018 with smaller differences between groups and a smaller body size in the heated group only after 15 days. Nevertheless, despite this ambiguity, our model showed that there was an overall statistically significant difference after 15 days of the nestling period. It is notable that individual variation and the treatment effect were most pronounced in 2017 (Figure 2c,d), when the external ambient temperatures were colder (Figure 1). One explanation for this could be that, even though the differences in nest temperature were similar (~2°C), there may have been larger and more variable differences in the microclimate elsewhere in the nest box itself when the ambient temperatures were lower. Another possible explanation could be that females had to brood more often and thus reduce the foraging provision of the nestlings. However, Álvarez and Barba (2014) did not find differences in the size or condition of the nestlings when females reduced its presence in the warmer nests. Between‐year differences in the mean tarsus length throughout the nestling period (up to and including day 15) could also be driven by food availability, perhaps with greater food availability in 2018 when ambient temperatures were greater (Figures 1, 2, 3).

Our general finding that nestlings from warmer nests fledge at a smaller body size than those from cooler nests may be related to James' rule (Blackburn et al., 1999), which proposes that body size should be larger in individuals living in cooler environments than those in warmer environments. The underlying rationale for this is that it is beneficial for individuals in colder environments to reduce their surface‐area‐to‐volume ratio to ameliorate heat loss (Teplitsky et al., 2008). Another explanation may be that nestlings under heat stress invest energy in thermoregulation at the expense of growth (as found by Andreasson et al., 2018). However, we believe this is unlikely in our case because our heated treatment was moderate (~2°C) compared to the heat shock imposed in their study (i.e., ~8–10°C higher than in our study).

It was perhaps a little surprising that the timing of hatching was not associated with tarsus length and body weight, as the relationship between hatching date and the availability of food (e.g., insects, especially caterpillars) is well‐established (Caro et al., 2013; Charmantier et al., 2008; Schaper et al., 2012; Tinbergen & Boerlijst, 1990; Visser et al., 2009). The lack of statistical significance in this particular case could be due to insufficient sample size to detect a small effect size or due to a stronger effect of temperature in the case of size measurements (tarsus length and body weight).

In addition, we would like to highlight the observation of an apparent reduction in fledging probability in the heated nests compared to the control group (Figure 2b) during 2018. This negative effect of heating has been observed in similar experiments, because temperatures inside the heated nests can exceed the optimal range of 12–31°C (Rodríguez & Barba, 2016b), causing a detrimental impact on nestling physiology and body condition (Belda et al., 1995; Salaberria et al., 2014). However, the temperatures reached in our study (~30–36°C) were somewhat lower than other studies (e.g., Andreasson et al., 2018; Rodríguez & Barba, 2016b) where there was no observed effect on the nestling survival. Therefore, even with higher temperature during 2018, it is unlikely that heat was the main driver of the fledging difference in our case. An alternative indirect explanation could be that parents reduce their attentiveness and feeding rates in heated nests leaving their offspring undernourished, but as we argue before, this seems unlikely because Álvarez and Barba (2014) found that feeding behavior did not change significantly in heated nests compared to control ones.

In summary, this study shows that an increase in nest temperature of around 2°C during the early development is sufficient to produce phenotypic changes in the individual of up to 1.6% in skeletal size at fledging and a reduction of 3.3% body weight. Although 1.5°C is the threshold agreed in the 2015 Paris Agreement (UNFCCC, 2015), other models suggest that global temperature surface will rise even more (Hughes, 2000; Kellstedt et al., 2008; Wormworth & Sekercioglu, 2011). It is clear that climate change and accompanying temperature increases could alter breeding success and the phenotypic development of nestlings. Even though cavity nests are by nature buffered against weather variation, their microclimate is strongly associated with ambient temperatures and they are thus not immune from the effects of climate change (Larson et al., 2018; Maziarz et al., 2017). These climate‐driven changes in body size could have further population‐level consequences later in the season because adult body size is an important predictor of adult survival, especially over winter (Rodríguez et al., 2016; Tinbergen & Boerlijst, 1990). Thus, a situation where increased spring temperatures leads to smaller body size at fledging (and presumably smaller adult size) could lead to reduced overwinter survival, even if winter temperatures are unaltered. In Europe, climate change is expected to bring both increased spring and summer temperatures, and decreased winter temperatures, and this could have an even larger negative effect on survival (Kelemen et al., 2009). Whatever the future may hold, understanding the relationship between weather and breeding success and development is crucial if we are to understand in greater detail the broader consequences of climate change on species.

## CONFLICT OF INTEREST

None declared.

## AUTHOR CONTRIBUTIONS

**Alejandro Corregidor‐Castro:** Data curation (equal); Formal analysis (equal); Investigation (equal); Writing‐original draft (equal); Writing‐review & editing (equal). **Owen R. Jones:** Formal analysis (equal); Funding acquisition (lead); Supervision (lead); Writing‐original draft (equal); Writing‐review & editing (equal).

## Supporting information

Figure S1

Figure S2

Figure S3

Appendix S1

## Data Availability

The data that support the findings of this study will be openly available in Zenodo (https://doi.org/10.5281/zenodo.4646978).
