# Supplementary figures and images for: The effect of nest temperature on growth and survival in juvenile Great Tits Parus major
Source: Ecol Evol. 2021 May 1;11(12):7346–53. doi: 10.1002/ece3.7565 (PMC8216922; doi:10.1002/ece3.7565)

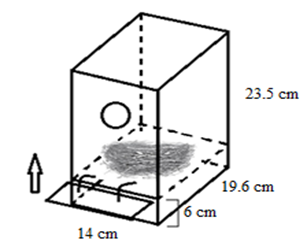

Supplement: Supplementary file 1 — Figure S1 [file ECE3-11-7346-s004.tiff]

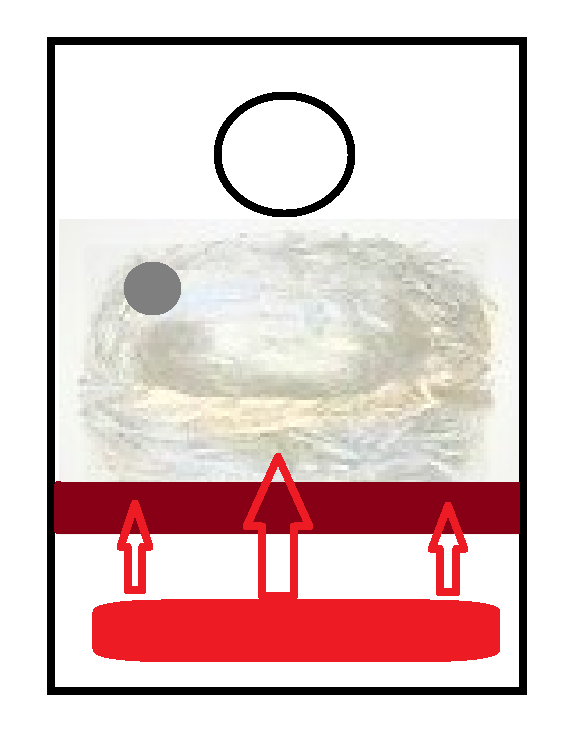

Supplement: Supplementary file 2 — Figure S2 [file ECE3-11-7346-s001.tiff]

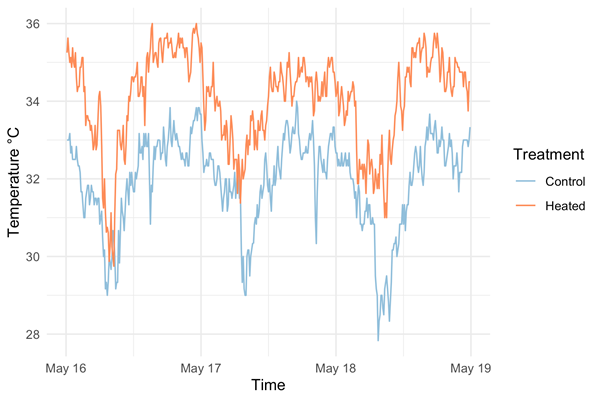

Supplement: Supplementary file 3 — Figure S3 [file ECE3-11-7346-s003.tiff]
