# Supplementary material for: The effect of nest temperature on growth and survival in juvenile Great Tits Parus major
Source: Ecol Evol. 2021 May 1;11(12):7346–53. doi: 10.1002/ece3.7565 (PMC8216922; doi:10.1002/ece3.7565)
Supplement: Supplementary file 4 — Appendix S1 [file ECE3-11-7346-s002.docx]

Appendix 1 – Additional details of experiment

## Schematic figure of the nestbox design

Our experiment used a wooden nest box designed as illustrated below (Figure S1). The box has a rectangular shape with a volume of approximately 6.5 litres (23.5 cm height and a 14 × 19.6 cm base). In order to replace the heating pack without disturbing the nest, we made a cut 6 cm above the base acting as a removable door, where we inserted a piece of polystyrene with a width of 2 cm. The placement of the polystyrene insulation left a chamber below the nest where we placed the heat packs. Over this material, we placed a water-resistant piece of wood with a thickness of 3 mm to protect the polystyrene from weathering.

*Figure S1 – an illustration of our nest box design, showing the location of the door that allows access to insert a heat pack.*

*Figure S2 – an illustration of our nest box design (from a frontal point of view), showing the location of the iButton.*

## Treatment temperature comparison

Here we show the temperature differences, and the absolute temperature values, of the two treatments in the study: heated boxes (in orange), and control boxes (in blue). Our comparison of nest temperature within heated and control nests showed that heated nests were, on average, 1.63^o^C (n=38) warmer than control nests.

*Figure S3 - Illustrative temperature record showing typical temperature differences between heated and control nest boxes over a 3 day period. The data were obtained at 10 minute intervals using iButton data loggers and the lines represent the mean of 3 (control) and 4 (heated) nest boxes.*

1. The Corresponding Author’s institution or funder has an existing agreement (pre-paid open access account) with Wiley and may pay the Article Publication Charge from this account on behalf of the author.

Click here to access the Account Code Finder Tool. Follow the directions on the page and enter the institution’s code in the field below.

Please enter institution code here:

0 OUT OF 4 CHARACTERS

Enter the Grant/Project Number if the institution or funder requires this information:

2. The Corresponding Author will pay directly or will arrange payment (request an invoice) for the Article Publication Charge. (Select from the dropdown menus below for any applicable discounts.)

3. The Corresponding Author is from a country on the Wiley Open Access Automatic Waiver List and requests an Automatic Waiver of the Article Publication Charge for this article.
